# Supplementary material for: Sequence-based virtual screening using transformers
Source: Nat Commun. 2025 Jul 28;16:6925. doi: 10.1038/s41467-025-61833-8 (PMC12304311; doi:10.1038/s41467-025-61833-8)
Supplement: Supplementary file 2 — Description of Additional Supplementary Files [file 41467_2025_61833_MOESM2_ESM.pdf]

Supplementary Data 1. PDBbind2020-subset dataset. This dataset comprises the training, validation, and test set split from PDBbind_10k. It includes columns for PDB ID, measured binding affinity in pKd units, protein sequences, and SMILES representations for the ligands.

Supplementary Data 2. $EGFR^{LTC}$-290 dataset. This dataset consists of inhibitors targeting $EGFR^{LTC}$, compiled from various literature sources. It provides SMILES representations for each inhibitor, alongside their measured IC_{50} values in nanomolar units. An additional "allosteric" column categorizes the inhibitor type, with '0', '1', and '0+1' denoting orthosteric, allosteric, and dual inhibitors, respectively.

Supplementary Data 3. TargetMol library for screening $EGFR^{LTC}$ inhibitors. This dataset lists the in-stock TargetMol library utilized for screening potential $EGFR^{LTC}$ inhibitors. It includes SMILES representations of the compounds and their original categories within TargetMol. Additionally, the dataset provides information on the maximum Tanimoto similarity for each compound compared to those in the $EGFR^{LTC}$-290 dataset.
